# Safety and Immunogenicity of an *In Vivo* Muscle Electroporation Delivery System for DNA-*hsp65* Tuberculosis Vaccine in Cynomolgus Monkeys

**DOI:** 10.3390/vaccines11121863

**Published:** 2023-12-18

**Authors:** Monique Ribeiro de Lima, Ana Cristina C. S. Leandro, Andreia Lamoglia de Souza, Marcio Mantuano Barradas, Eric Henrique Roma, Ana Teresa Gomes Fernandes, Gabrielle Galdino-Silva, Joyce Katiuccia M. Ramos Carvalho, Renato Sergio Marchevsky, Janice M. C. Oliveira Coelho, Eduardo Dantas Casillo Gonçalves, John L. VandeBerg, Celio Lopes Silva, Maria da Gloria Bonecini-Almeida

**Affiliations:** 1 Laboratory of Immunology and Immunogenetic in Infectious Diseases, Instituto Nacional de Infectologia Evandro Chagas, Fundação Oswaldo Cruz, Rio de Janeiro 21040-360, RJ, Brazil; nickrlima@gmail.com (M.R.d.L.); ana.leandro@utrgv.edu (A.C.C.S.L.); andreia.lamoglia@ini.fiocruz.br (A.L.d.S.); marcio.barradas@ioc.fiocruz.br (M.M.B.); eric.roma@ini.fiocruz.br (E.H.R.); ana.fernandes@ini.fiocruz.br (A.T.G.F.); gabrielle.galdino@ini.fiocruz.br (G.G.-S.); joyce@ucdb.br (J.K.M.R.C.); 2 Division of Human Genetics, South Texas Diabetes and Obesity Institute, The University of Texas Rio Grande Valley, Brownsville, TX 78520, USA; john.vandeberg@utrgv.edu; 3 Laboratory of Neurovirulence, Instituto de Biotecnologia em Imunobiológicos, Biomanguinhos, Fundação Oswaldo Cruz, Rio de Janeiro 21040-360, RJ, Brazil; march@bio.fiocruz.br; 4 Laboratory of Pathology, Instituto Nacional de Infectologia Evandro Chagas, Fundação Oswaldo Cruz, Rio de Janeiro 21040-360, RJ, Brazil; janice.coelho@ini.fiocruz.br; 5 Farmacore Biotecnologia Ltda, Ribeirão Preto 14056-680, SP, Brazil; eduardo@easytechls.com.br (E.D.C.G.); clsilva@fmrp.usp.br (C.L.S.); 6 Laboratory for Research and Development of Immunobiologicals, School of Medicine of Ribeirão Preto, University of São Paulo, Ribeirão Preto 14049-900, SP, Brazil

**Keywords:** tuberculosis, electroporation, vaccine, DNA-hsp65, safety, immunogenicity, nonhuman primate

## Abstract

A Bacille Calmette–Guérin (BCG) is still the only licensed vaccine for the prevention of tuberculosis, providing limited protection against Mycobacterium tuberculosis infection in adulthood. New advances in the delivery of DNA vaccines by electroporation have been made in the past decade. We evaluated the safety and immunogenicity of the DNA-hsp65 vaccine administered by intramuscular electroporation (EP) in cynomolgus macaques. Animals received three doses of DNA-hsp65 at 30-day intervals. We demonstrated that intramuscular electroporated DNA-hsp65 vaccine immunization of cynomolgus macaques was safe, and there were no vaccine-related effects on hematological, renal, or hepatic profiles, compared to the pre-vaccination parameters. No tuberculin skin test conversion nor lung X-ray alteration was identified. Further, low and transient peripheral cellular immune response and cytokine expression were observed, primarily after the third dose of the DNA-hsp65 vaccine. Electroporated DNA-hsp65 vaccination is safe but provides limited enhancement of peripheral cellular immune responses. Preclinical vaccine trials with DNA-hsp65 delivered via EP may include a combination of plasmid cytokine adjuvant and/or protein prime–boost regimen, to help the induction of a stronger cellular immune response.

## 1. Introduction

Pulmonary tuberculosis (TB) caused by *Mycobacterium tuberculosis* remains one of the leading worldwide killers among all infectious diseases, despite extensive use of the *Mycobacterium bovis* bacilli Calmette–Guérin (BCG) vaccine. Ten million infected people developed active disease and 1.6 million died from the disease in 2021 [1]. A new TB vaccine is urgently needed to replace or to be used as a booster for the BCG vaccine. Among the novel vaccine strategies, plasmid DNA-based TB vaccines have received increasing attention because of their safety and induction of both CD4^+^ and CD8^+^ T cell responses. One of the main products under development by our group is a DNA vaccine containing the gene that expresses the *Mycobacterium leprae* hsp65 antigen (DNA-*hsp65*) with broad immunomodulatory and/or immunoregulatory properties [2]. Our previous studies have demonstrated that DNA-*hsp65* confers a high level of protection in mice both prophylactically and therapeutically [2]: it works in prevention; can be used as a BCG booster [3]; can be used alone or in association with antimycobacterial drugs to completely control chronic TB, multidrug-resistant (MDR)-TB and latent TB (LTB) infection; and can prevent reactivation of infection [4,5,6,7].

Since the discovery that DNA-*hsp65* can have immunomodulatory and/or immunoregulatory action against TB, several other investigations have been conducted by our group with other infectious diseases, autoimmune diseases, allergy diseases, and tumors. Thus, as reviewed in reference [2], it was demonstrated that DNA-*hsp65* presents broad immunotherapeutic properties for infectious diseases such as leishmaniasis, schistosomiasis, paracoccidioidomycosis, chromoblastomycosis, helminth infections; autoimmune diseases such as diabetes, arthritis, encephalomyelitis, and atherosclerosis; allergy diseases such as asthma and atopic dermatitis; and tumors in experimental models of mice and in phase I/II clinical trials in humans. Thus, DNA-*hsp65* actively participates in the activation of innate immunity, acting as an endogenous adjuvant and playing a fundamental role in the activation and control of adaptive immunity [4,5,6,7].

Studies have shown that DNA-*hsp65* preventive and/or therapeutic activities [2] are associated with *in vitro* activation of human dendritic cells (DCs) and macrophages [4,6]; stimulation of CD4 and CD8 [8] and B lymphocytes; induction of murine Th1, Th17, and Treg pattern of immune response [9,10]; production of cytokines and activation molecules necessary to restrict bacterial growth [4,9,10,11]. The protection conferred in animals previously vaccinated with DNA-hsp65 and subsequently challenged with *M. tuberculosis* was associated with the induction of CD8^+^CD44^hi^IFN-γ^+^ and CD4^+^CD44^hi^IFN-γ^+^ phenotype cells, while in animals vaccinated with BCG, the cells were characterized as CD4^+^CD44^lo^IFN-γ^+^. In addition, BCG prime and DNA*hsp65* boosters studies improved immunogenicity and efficacy by comparison with vaccination with BCG or DNA-*hsp65* alone [12]. These results show that the prime–boost strategy is an important alternative to induce effective protection against TB. Furthermore, B cells are also activated after vaccination with DNA-*hsp65* and modulate the formation of memory CD8^+^T cells after the challenge of mice with *M. tuberculosis* [10,11,12].

Although vaccination with naked DNA has a high immunostimulatory potential for both innate and adaptive immunity, the immune response can be increased with the use of adjuvants. For instance, lipidic emulsions, poly lactic-co-glycolic acid (PLGA) microsphere, and liposome formulations as adjuvant delivery systems indeed showed a significant enhancement of the efficacy of the vaccine in response to challenge with *M. tuberculosis* in a murine model.

A number of other immunogenic *M. tuberculosis* antigens, such as rv2190c [13], rv1733c [14], Ag85A [15,16], Ag85B [17,18], Mtb32C [19], and fusion proteins such as HspX-PPE44-EsxV [20] or ESAT6/Ag85A [21] have been expressed in plasmid DNA vectors.

It is very well established that DNA vaccines for TB stimulate cell-mediated immunity by activating CD4^+^ and CD8^+^ T cells. However, DNA vaccines have low cell transfection efficiency in vaccinated animals and require the use of repeated intramuscular or subcutaneous injections. Due to these drawbacks, efforts are being made to increase the immunogenicity of DNA vaccines by improving the DNA delivery techniques. One approach is to increase the efficiency of plasmid-based DNA vaccination by electroporation (EP). *In vivo* EP is an efficient vaccine strategy that enhances cell permeability, local tissue inflammation, and immunogenicity in experimental animals [22,23,24,25,26,27] and in humans [28,29,30,31]. When EP is applied to the target organism, the encoded antigens are expressed and elicit the corresponding immune response, with the potential to induce antibody-mediated, helper T cell-mediated, and cytotoxic T cell-mediated immune responses [32]. The TB DNA vaccine administered by EP has been tested in mice [33,34,35], pigs [22], and non-human primates [36,37]. As far as we know, an EP-DNA vaccine against tuberculosis has not yet been tested in humans. Our goal was to determine the safety and immunogenicity induced by the *hsp65-*DNA vaccine delivered by muscular EP in cynomolgus macaques (*Macaca fascicularis*), a nonhuman primate model that closely resembles humans in clinical and immune response characteristics of *M. tuberculosis* infection.

## 2. Materials and Methods

### 2.1. Animal Model and Medical History

Animal selection, quarantine before vaccination, and medical history: Twenty-one male adult cynomolgus macaques, ranging from 7 to 10 years in age and weighing 6.0 to 7.6 kg were selected from the Animal Breeding Center from the Institute of Science and Technology in Biomodels, Oswaldo Cruz Foundation (Fiocruz), Rio de Janeiro, Brazil. The Oswaldo Cruz Foundation Animal Care and Use Committee (IACUC/Fiocruz) approved the project (protocol number PO184-03).

Serologic screening was conducted for IgG and IgM anti-cytomegalovirus (CMV), toxoplasmosis, hepatitis B (HBV), and *Trypanosoma cruzi* (Chagas disease) infection, and clinical chemistry, hematological and immunological profiles were established. Nine of the twenty-one animals were excluded from the study because they were IgG-positive for CMV and/or toxoplasmosis. Animals were housed in individual cages for a total of twelve months (four before vaccination and six after the first dose of the vaccine). They were housed under controlled conditions of temperature, light (12 h light/12 h dark cycles), and humidity. Food and water were available ad libitum. Animals were monitored at least twice daily and fed commercial monkey chow, and seasonal fruit once a day by trained personnel. Environmental enrichment consisted of toys and balls filled with treats, visual (TV and mirrors), and audio enrichment.

Behavior histories were assessed daily, three to four months before and after vaccination to ensure absence of any abnormality.

The animals were sedated by intramuscular injection of 5% (10 mg/kg) ketamine hydrochloride. Blood samples were collected via femoral venipuncture into vacutainer tubes.

Animals were subjected to X-ray analysis 30–45 days before the first vaccination and after the last vaccination.

Tuberculin skin test (TST) analyses were performed twice before and four months after the first vaccine dose using 0.1 mL of mammalian old tuberculin via intradermal palpebral skin test (Synbiotics, San Diego, CA, USA). Palpebral reactions were graded at 48 and 72 h with the standard 1 to 5 scoring system, as previously defined [38]. Grade 0—no cutaneous reaction; grade 1—bruise, with extravasation of blood in the eyelid; grade 2—palpebral erythema with minimal swelling; grade 3—moderate swelling with or without erythema; grade 4—well-defined palpebral swelling with drooping and presence of erythema; and grade 5—necrosis and marked swelling with eyelid closed or partially closed. Grades interpretation: grades 0, 1, and 2 were considered to be TST negative; grades 3, 4, and 5 were considered to be TST positive.

### 2.2. Vaccine Schedule and Electroporation

**Figure 1:**
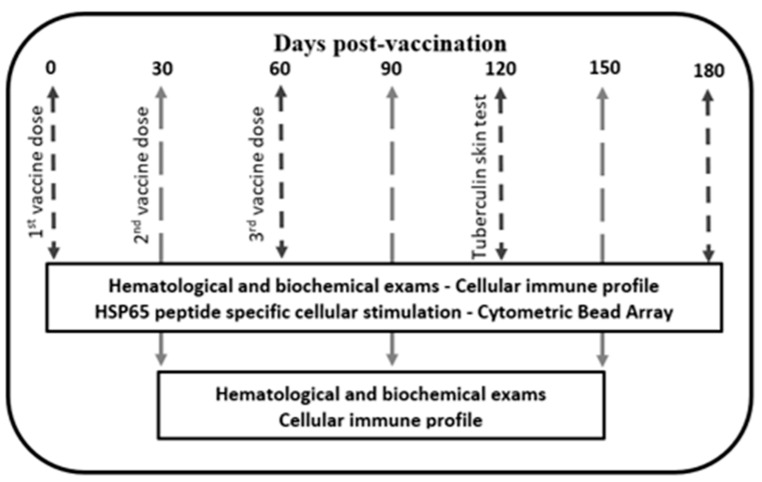
Vaccination scheme. Cynomolgus macaques were immunized with electroporated DNA-*hsp65* vaccine delivered in three doses at 30-day intervals by *in vivo* muscular electroporation. Clinical, hematological, biochemical, and immunological profiles were evaluated as represented.

Vaccination was performed by injection of 1000 μg pVAX-*hsp65-DNA* in 1 mL of plasmid DNA diluted in saline into the quadriceps muscle of eight monkeys. The injection site was subject to EP using equipment (Inovio Biomedical Co., San Diego, CA, USA) adjusted according to the following parameters: time = 60; Ntrain = 1; Nsequence = 2; volt = 200. The animals from control group (n = 4) were immunized with an empty plasmid DNA (DNA-pVAX) and subjected to the same EP protocol. Animals were boosted at four-week intervals for three vaccinations. The vaccination scheme and laboratory tests are represented in Figure 1.

### 2.3. Genetic Vaccine Construction

The DNA-*hsp65* vaccine was constructed by inserting the gene that expresses the *M. leprae* hsp65 protein into the cloning site of the *pVAX* plasmid (Invitrogen^®^, Carlsbad, CA, USA) previously digested with the restriction enzymes *Bam* HI and *Not I* (Gibco BRL, Gaithersburg, MD, USA). The empty vector DNA-*pVAX*, without the *hsp65* gene, was used as a control. DNA-*hsp65* and DNA-*pVAX* used for vaccinations were produced in *Escherichia coli* transformed with the respective plasmids and cultivated in LB liquid medium (Gibco BRL) containing 100 µg/mL of ampicillin. The plasmids were then purified using the Concert High Purity Maxiprep System (Gibco BRL). The Gene Quant II apparatus (Pharmacia Biotech, Buckinghamshire, UK) was used to evaluate the concentration of plasmids in the samples.

### 2.4. Clinical Signs and Vaccine Safety

The animals were assessed daily to identify changes in behavior, such as appetite loss, refusal to receive snacks, apathy or aggressiveness; and signs such as local pain, itching, redness, and swelling. Systemic analysis was conducted monthly, including fever, weight loss, bruises, anemia, red and white cell counts, and renal and hepatic enzyme alteration.

### 2.5. Cellular Immune Profile

Whole blood and specific antibody mixtures were incubated in the dark at room temperature for 30 min. Then, an FACS™ Lysing solution (Beckson-Dickinson, BD, Franklin Lakes, NJ, USA) was added, and the mixture was gently homogenized. After incubation for 15 min, samples were washed with 4 mL of cold phosphate-buffered saline (PBS), pH 7.2. At the end of the protocol, 300 μL of 2% paraformaldehyde solution in PBS, pH 7.2, was added. Final volume was adjusted with 800 μL PBS, pH 7.2. Data acquisition was performed using a Dako Cyan ADP flow cytometer, and results were analyzed with Summit V4.3 (Dako Colorado, Santa Clara, CA, USA). The following fluorochrome-conjugated anti-human monoclonal antibodies (MAb) that cross-react with cynomolgus monkey cellular antigens were used (BD Pharmingen, San Diego, CA, USA): CD3 APC-Cy7 or FITC (SP34), CD4 APC or PE-Cy7 (L200), CD8 PercP-Cy5 (SK1), CD11c (S-HCL-3), CD14 FITC (M5E2), CD16 ECD or FITC (3G8), CD20 PercP (L27) or FITC (2H7), CD28 APC (CD28.2), CD95 PE (DX2), CD123 PE (7G3) and HLA-DR ECD (Immu 357).

Negative lineage (Lin^−^) CD3^−^/CD14^−^/CD16^−^/CD20^−^ was gated to characterize myeloid dendritic cells (DCs)—mDCs (Lin^−^, HLA-DR^+^/CD11c^+^/CD123^−^) and plasmacytoid—pDCs (Lin^−^, HLA-DR^+^/CD11c^−^/CD123^+^) phenotypes. Central memory (CM, CD3^+^/CD4^+^ or CD3^+^/CD4^−^ and CD28^+^/CD95^+^) and effector memory (EM., CD3^+^/CD4^+^ or CD3^+^/CD4^−^ and CD28^−^/CD95^+^) T lymphocytes subpopulations were analyzed.

### 2.6. HSP65 Peptide Specific Cellular Stimulation

Freshly isolated PBMC were incubated with monoclonal anti-human CD28 (clone 28.2; BD) and CD49d (clone 9F10; BD), each at a final concentration of 10 µg/mL, in tubes slanted 5° from horizontal at 37 °C with 5% CO_2,_ as described by Gauduin et al., 2004 [39].

After this first stimulus, cells (1 × 10^6^) were washed and stimulated with 10 µg/mL of 105 HSP65 (15 to 20 mer overlapping AA) peptides pool (PEPscreen^®^, Sigma-Aldrich, St. Louis, MO, USA; Supplemental Table S1). The internal controls of the experiment included stimulation with a combination of 100 ng/mL of both Staphylococcal enterotoxin A and B (SEA and SEB, Sigma, St. Louis, MO, USA) or RPMI medium. Cells were incubated for 16 h at 37 °C in a humidity chamber. Supernatants were recovered for Th1 and Th2 cytokine stimulation. The cells were resuspended in 300 µL RPMI and 10 µg/mL of Brefeldin A (Sigma). After 6 h of incubation, cells were recovered, washed, and incubated at 4 °C for 30 min with the following antibodies for surface markers with anti-human CD3 APC-Cy7 (SP34), CD4 APC (L200), CD8 PercP-Cy5 (SK1), CD14 FITC (M5E2) and CD69 PE (FN50) from BD (USA); CD16 ECD (3G8) and CD20 APC (B9E9) from Beckman-Coulter (USA). Cells were fixed using Cytofix (BD), washed, and permeabilized by Permwash (BD). Cells were stained at 4 °C for 30 min with intracellular markers with anti-human IFN-γ FITC (B27), TNF-α PE (MAb11) and Ki-67 FITC (B56); anti-IL-10 PE (JES3-9D7) and Granzyme B APC (GB12) from Caltag-Life Technology (USA), IL-12 FITC (MT618) and Perforin FITC (Pf-344) (Mabtech Inc., Cincinnati, OH, USA). Data acquisition was performed using a Dako Cyan ADP flow cytometer, and results were analyzed with Summit V4.3 (Dako).

### 2.7. Th1 and Th2 Cytokines Induced by EP-pVAX-hsp65-DNA Vaccine

The cultured cell supernatants collected just before Brefeldin A administration were stored frozen at −80 °C. A Non-Human Primate Th1/Th2 Cytokine Kit (Cytometric Bead Array, BD Pharmingen) was used to quantify IFN-γ, TNF-α, IL-2, IL-4, IL-5 and IL-6 cytokines. Positive and negative internal controls were cultured cells stimulated with and without SEA and SEB. The samples were thawed and centrifuged at 5000× *g* for 5 min at 4 °C to remove any precipitate. The supernatants were then incubated in darkness with the microspheres for 3 h at room temperature and washed. Data acquisition was performed using a Dako Cyan ADP flow cytometer and results were analyzed with Summit V4.3 (Dako).

### 2.8. Muscle and Lung Histopathology

Samples of right and left quadriceps muscle, removed from the EP injection site, and right and left upper, middle, and lower lung lobe tissues samples were collected and maintained in 10% neutral buffered formalin or in Tissue-Tek optimum cutting temperature (OCT, Sakura, Los Angeles, CA, USA) at room temperature or at −80 °C, respectively. Tissues were sectioned and stained by hematoxylin and eosin. Histopathology analysis was performed to identify tissue alterations and infiltration of inflammatory cells, particularly lymphocytes and macrophages.

### 2.9. Outcomes

The primary safety outcome was determined by changes in laboratory (hematology, renal and hepatic values), behavioral, and physical parameters. The secondary outcome was determined by the increase in peripheral immune cells and their activation and the increase in cytotoxic markers (CD69^+^, perforin, and granzyme) and cytokines (mainly IFN-γ and TNF-α) during the post-vaccination period.

### 2.10. Statistical Analysis

Statistical analysis was carried out with Prism 9.0 software (Graph-Pad, San Diego, CA, USA). The two-way ANOVA with the Sidak correction was used to compare DNA-*hsp65* vaccinated and control group. The Sidak multiple comparisons test was used to compare statistical differences between the groups for each time point evaluated, before and after vaccination. Paired intragroup comparisons were used to analyze statistical differences among time points. A *p* value of <0.05 was considered significant. Clinical, biochemistry, hematological, and immunological data were analyzed as follows: (i) data from the DNA*-hsp65* vaccinated animals were compared with their own baseline data by paired T-test analysis, and (ii) data from the EP-*pVAX-hsp65*-DNA vaccinated group was compared with the control group (intergroup unpaired analysis).

## 3. Results

### 3.1. Animal Preclinical Parameters

Clinical data were collected over 12 months. All of the 21 selected animals were IgM negative for CMV, toxoplasmosis, *Trypanosoma cruzi,* and HBV infection, and 12 of them were IgG negative. The other nine animals were IgG-positive for CMV and/or toxoplasmosis and were excluded from the study. All laboratory results were within the normal parameters described by the Center for Nonhuman Primates of the Institute of Science and Technology in Biomodels, Oswaldo Cruz Foundation, for male and age-matched cynomolgus macaques. The animals that were identified during the pre-vaccination period to harbor intestinal parasites (such as *Blastocystis hominis*, *Balantidium coli*, *Iodomoeba butschlii*, *Entamoeba histolytica* and *E. coli*) were treated with 90 mg/kg of secnidazol. The TST was negative (grade zero), and no pulmonary abnormalities were identified in the X-ray analysis.

### 3.2. DNA-hsp65 Vaccine Is Safe with No Adverse Events

Twelve monkeys were vaccinated and followed for six months. None of the vaccinated animals showed cutaneous alterations at the vaccine administration site or at any other body site, nor changes in body weight, temperature, food consumption, behavior, morbidity, or mortality during the entire experiment. There were no vaccine-related effects on hematological profiles (Supplemental Table S2) or renal and hepatic profiles (Supplemental Table S3) compared to the pre-vaccination parameters.

### 3.3. DNA-hsp65 Vaccine Did Not Induce Tuberculin Skin Test Conversion or Pulmonary X-ray Alteration

**Figure 2:**
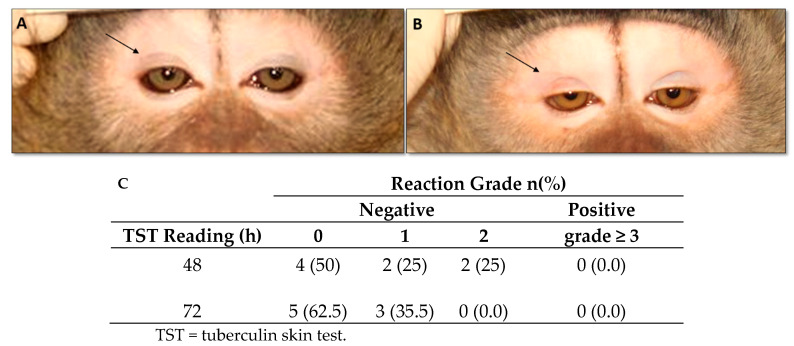
Tuberculin skin test. Cynomolgus macaques were immunized with electroporated DNA-*hsp65* vaccine delivered in three doses at 30-day intervals by *in vivo* muscular electroporation. Reaction to the tuberculin skin test was performed 30 days after the last dose of the vaccine. (**A**) No reaction (grade zero), (**B**) bruises (grade 1), and (**C**) number and frequency of reactive tuberculin skin tests.

Three doses of DNA*-hsp65* vaccine did not result in TST conversion. Five (62.5%) of the eight vaccinated animals showed no reaction (TST grade zero, Figure 2A), and three showed only the presence of bruises (TST grade 1, Figure 2B) at 72 h. Two of the four control animals displayed erythema without edema (TST grade 2), and the other two displayed only bruises (TST grade 1) at the site of injection. Pulmonary X-rays were taken 30 days after the third vaccine dose, and no alterations were identified in vaccinated or control animals. 

### 3.4. Peripheral Cell Immune Profile and Activation Markers Induced by DNA-hsp65 Vaccine

**Figure 3:**
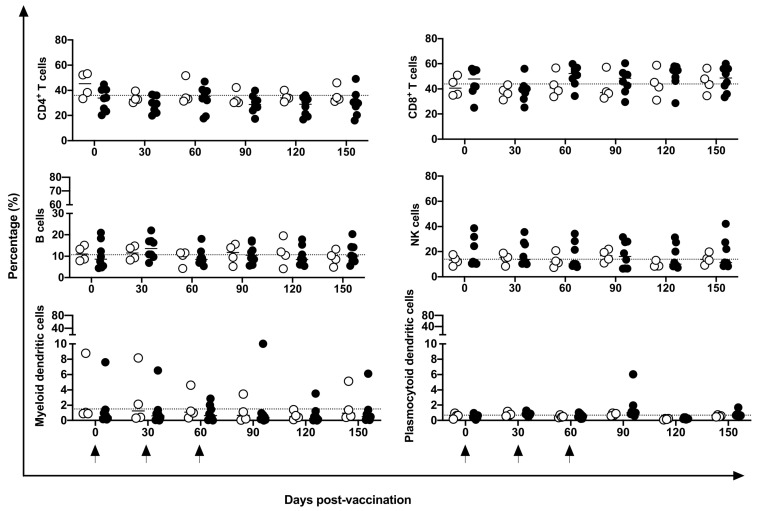
Frequency of immune cells in the peripheral blood. Frequency of CD4^+^ and CD8^+^ T cells, B cells, NK cells, myeloid dendritic and plasmocytoid dendritic cells in unvaccinated (open circles) and electroporated DNA-*hsp65* vaccinated (black circles) cynomolgus macaques. Arrows represent the three doses of vaccine.

**Figure 4:**
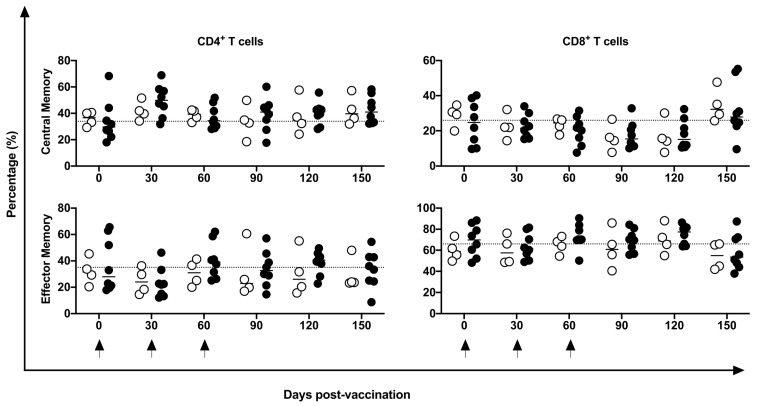
Frequency of central and effector memory cells in the peripheral blood. Frequency of CD4^+^ and CD8^+^ T CM and EF cells in unvaccinated (open circles) and electroporated DNA-*hsp65* vaccinated (black circles) cynomolgus macaques. Arrows represent the three doses of vaccine.

**Figure 5:**
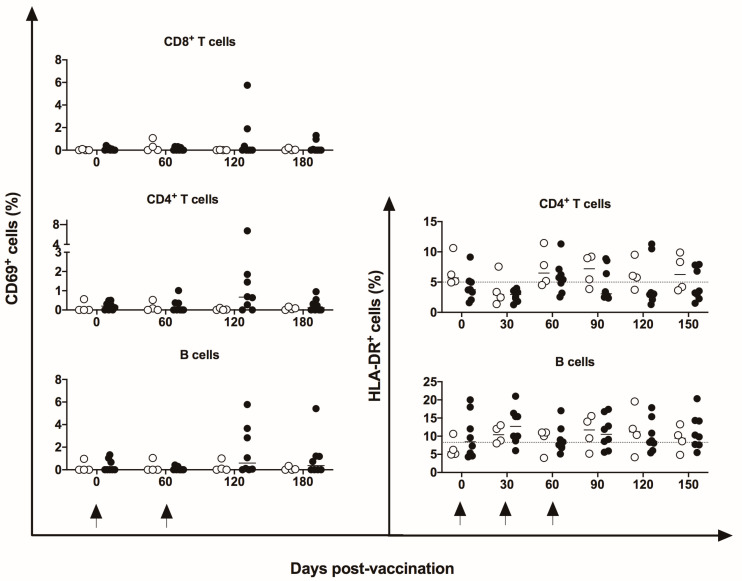
Frequency of activation marker CD69^+^ in peripheral blood. Frequency of CD4^+^/CD69^+^, CD8^+^/CD69^+^, and CD19^+^/CD69^+^ cells in unvaccinated (open circles) and electroporated DNA-*hsp65* vaccinated (black circles) cynomolgus macaques. Arrows represent the three doses of vaccine.

No differences were identified in the frequency of peripheral CD4^+^ and CD8^+^ T, B, or NK cells nor in mDCs and pDCs (Figure 3). Indeed, in five of eight vaccinated animals, an increase in CM CD4^+^ T cells at 30 days post-vaccination period was observed compared to baseline data; however, no statistical difference was demonstrated, perhaps because of the large variation in baseline values (19.2 to 74%). No changes were induced in the CM or EM T CD8^+^ cells (Figure 4) by the DNA*-hsp65* vaccine. The expression of the CD69^+^ activation marker in CD8^+^, CD4^+^T, and B cells increased transitorily in three of the eight vaccinated animals, with no statistical difference at 120 days post-vaccination (Figure 5). No changes in the HLA-DR^+^ expressing CD4^+^ T and B cells were observed (Figure 5).

### 3.5. Proliferative, Lytic, and Apoptotic Markers Induced by DNA-hsp65 Vaccine

**Figure 6:**
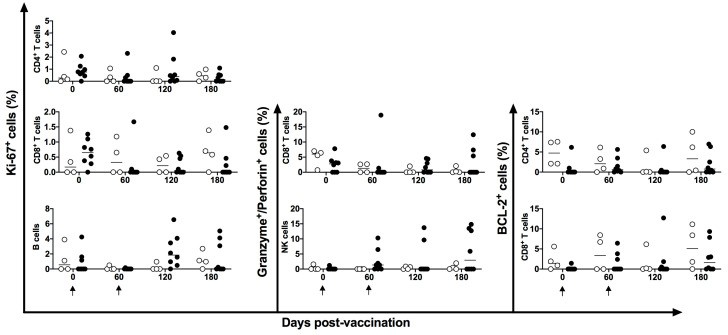
Frequency of proliferation, lytic and apoptotic markers in peripheral blood. Frequency of CD4^+^, CD8^+^, and CD19^+^ cells expressing Ki67^+^, BCL2^+^, and granzyme/perforin markers in unvaccinated (open circles) and electroporated DNA-*hsp65* vaccinated (black circles) cynomolgus macaques. Arrows represent the three doses of vaccine.

There was no alteration in the intracellular expression of the Ki-67^+^ marker in either T or B cells. However, Ki-67^+^ showed augmented expression on B cells in four of the eight vaccinated animals at 120 days post-vaccination. Granzyme B^+^ and perforin^+^ granules and BCL-2^+^ markers showed no statistical difference after stimulation with a pool of HSP65 peptides in DNA-*hsp65* vaccinated animals (Figure 6).

### 3.6. Cytokines Induced by DNA-hsp65 Vaccine

**Figure 7:**
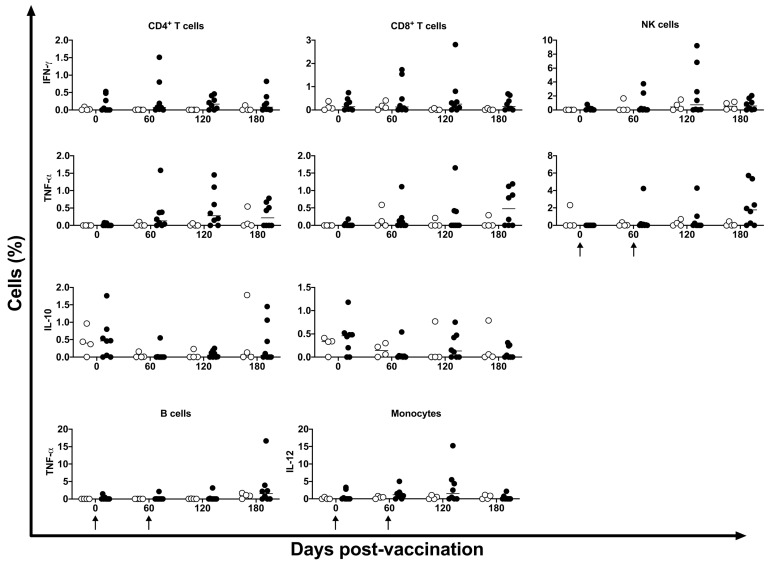
De novo cytokine expression in immune cells. Expression of IFN-γ, TNF-α, IL10, and IL-12 in CD4^+^ and CD8^+^ T cells, NK cells, and CD19^+^ and CD14^+^ cells was identified after stimulus of the cells with HSP65 peptide pool in unvaccinated (open circles) and electroporated DNA-*hsp65* vaccinated (black circles) cynomolgus macaques. Arrows represent the three doses of vaccine.

De novo cytokine modulation (IFN-γ, TNF-α, IL-10, and IL-12) was assessed by intracellular flow cytometry at 0, 60, 120, and 180 days post DNA*-hsp65* vaccination. No statistical differences were observed among the time points. Two of the eight animals showed increased CD4^+^, CD8^+^ T, and NK cells expressing IFN-γ at 60 and 120 days post-vaccination. CD4^+^ T cells expressing TNF-α were increased in four of eight vaccinated animals at 120 days post-vaccination, and CD8^+^ T and NK cells were increased in four of eight vaccinated animals at 180 days post-vaccination (Figure 7). This transient increase in cytokine expression occurred after the third dose of the vaccine.

**Figure 8:**
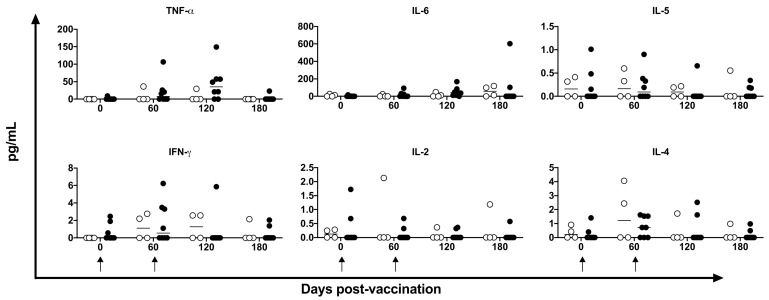
De novo cytokines quantification in supernatant. TNF-α, IL-6, IL-5, IFN-γ, IL-2, and IL-4 were measured in the supernatant of stimulated cell culture with HSP65 peptides pool by CBA in unvaccinated (open circles) and electroporated DNA-*hsp65* vaccinated (black circles) cynomolgus macaques. Arrows represent the three doses of vaccine.

The supernatants of the PBMC cultures were assayed for the Th1/Th2 profile using a non-human primate CBA kit. No statistically significant difference was observed after a two-way ANOVA test at any time point for any cytokine evaluated (Figure 8). However, the level of TNF-α was higher in four of the eight vaccinated animals at 120 days post-vaccination. Although the mean level was 6.01-fold higher in vaccinated animals (44.32 ± 48.47 pg/mL) compared to unvaccinated animals (7.37 ± 14.32 pg/mL), there was a large within-group difference. The IL-6 levels showed a similar profile to the TNF-α profile.

### 3.7. Muscle and Lung Histopathological Analysis

Histopathology analysis of all lung sections presented no structural alterations or relevant inflammatory cell infiltration. Cases of anthracosis were observed, but they were not related to vaccine administration since the condition was in control as well as vaccinated animals. A focal and sparse mononuclear inflammatory process was seen in both sides of the quadriceps muscle tissue mainly between muscle fibers and in the conjunctive tissue, without fibrinoid necrosis. This feature was observed in all vaccinated animals (Supplemental Figure S1).

## 4. Discussion

Previously, we demonstrated that DNA-*hsp65* vaccination confers a high level of protection in mice both prophylactically and therapeutically [2]: in heavily infected mice, simply by giving DNA-*hsp65* immunotherapy, the immune response can be caused to switch from one that is relatively inefficient and gives bacterial stasis (Th2) to one that kills the bacteria (Th1), and persistent bacteria can be eliminated [11,40]. Here we report the results of the first test of DNA-*hsp65* immunogenicity and safety in a primate species using an EP-assisted delivery system DNA vaccine.

Enhancement of naked DNA vaccination through EP has emerged as a new technology to increase the efficacy of DNA vaccine administration and has been shown to be safe, tolerable, and acceptable in most healthy human trial participants [31,41,42,43,44].

Cynomolgus macaques have been extensively used as an animal model in several preclinical vaccine trials, using an EP-assisted delivery system, including DNA targets against hemorrhagic fever virus [45], Lassa virus [46] Ebola [47], Marburg virus [48], SHIV [49], Venezuelan equine encephalitis virus [50], and HIV [51]. The results of this first preclinical trial demonstrate that the DNA-*hsp65* vaccine administered by muscular injection through EP is safe and well tolerated in cynomolgus macaques. No significant differences in local or systemic parameters were observed between vaccinated and control animals at a dose of 1000 µg DNA. None of the animals showed reactional lesions or behavioral changes indicative of local discomfort or pain, or abnormal systemic alterations related to vaccination, suggesting that this vaccination scheme is likely to be safe for advancement to a clinical trial. None of the DNA-*hsp65* vaccinated animals converted the TST: this result suggests that it will be possible to distinguish between people vaccinated with this vaccine and those with latent or active *M. tuberculosis* infection.

Naked plasmid DNA vaccination induces a poor immune response in large animal models, including nonhuman primates, as well as in humans. Previous results using HIV-1 Gag DNA vaccine with or without IL-12 and/or IL-15 plasmid adjuvant did not induce strong cellular immunity in healthy human subjects [52].

The primary concept for EP-derived vaccination is the uptake of DNA into various cell types, such as subcutaneous, muscle, and dendritic cells, in which it reaches the nucleus and initiates gene transcription, protein production, and post-translational modifications. New exogenous proteins formed are presented in the context of HLA class I and II molecules [32], resulting in an expected increase in a specific immune response. In our NHP model, the DNA-*hsp65* vaccination system induced transient cellular immunogenicity in peripheral blood. To our knowledge, only one previous report described the immunogenicity or efficacy of an EP-DNA vaccine against tuberculosis in NHP. In that report, three doses (0.5 mg) of EP and non-EP *Ag85A/ESAT6* DNA vaccine induced equal amounts of specific antibodies in rhesus macaques. After a booster with both Ag85A and ESAT6 proteins, the levels of antibodies increased 7–8 times [36]. The same profile was seen in rhesus macaques vaccinated with a cocktail of EP-Gag/Env-DNA vaccine; where the frequencies of granzyme B^+^ and CD4^+^ and CD8^+^ T cells expressing IFN-γ^+^ were low (0.02–0.08%), even after one year post-vaccination [53]. An increment of humoral immune response to this vaccine prototype was achieved when the animals were boosted with both Gag/Env proteins [37]. No differences in the frequency of CD4^+^ and CD8^+^ T cells, or the activation marker CD69^+^, or even in the CM, EM, or naïve cells in the peripheral blood were observed in rhesus macaques vaccinated with a *Mtb* mutant in SigH (MtbΔ*sigH*) vaccine through the mucosal route [54]. Future studies using the BCG vaccine as a prime and DNA-*hsp65* as a booster may enhance the peripheral immune response of the DNA-*hsp65* in the NHP model.

In the tuberculosis vaccine pipeline, the candidate M72/AS01_E_ formulation has proceeded to several clinical trials. A phase II, double-blind randomized, controlled clinical trial conducted in India proved its immunogenicity for up to three years. The analysis showed no specific CD8+ T cell response after vaccination, as we observed in our pre-clinical model. Regarding the specific CD4+ T cells, the frequency dropped from seven months post-vaccination to the three-year endpoint of the study. The frequency of cells expressing at least two cytokines was 0.15% [55]. In our study, only CD4^+^ and CD8^+^ T cells expressing TNF-α were reported transitorily at 120 days post-vaccination in 50% of the vaccinated animals. IFN-γ is a critical cytokine for the control of *M. tuberculosis* infection; however, its correlation with vaccine protection in NHP is controversial [56].

Although it is widely accepted that CD4^+^ T cells play an essential role in resistance to *M. tuberculosis* infection in experimental animals, their role is still unclear in human latent tuberculosis infection and disease. The absence of a high number of CD4^+^ T cells in our study does not mean that the animals were not efficiently immunized. Even at the site of infection, CD4^+^ T cells do not have an abundant profile. In an intravital image approach, reduced specific T cells and low cytokine secretion were not markedly present within the granuloma [57]. Our knowledge of the role of CD4^+^ T cells in the control of *M. tuberculosis* infection, in the peripheral blood as well as at the site of infection, is still incomplete. On the other hand, many *in vivo* and *in vitro* studies have provided evidence of the significant role of CD8^+^ T cells in the control of the disease [58]. In our study, six of eight immunized animals showed a slight increase above the normal range in frequency of CD8^+^ T cells at 120 days after DNA-*hsp65* vaccination. This transient increase might reflect the presence of EM CD8^+^ T cells in the vaccinated animals. The large variation in levels of these cells in the vaccinated animals prevents the identification of subtle changes in these populations. Therefore, the absence of significant differences prevents us from reaching a clear conclusion about the role of these cells in DNA-*hsp65* vaccination. This transient increase might reflect the posterior migration of CD8^+^ T cells in the vaccinated animals. Differences in activation markers in lung resident and peripheral CD4^+^ and CD8^+^ T cells were reported previously by Sallin et al. [59] and reviewed by Lewinsohn and Lewinsohn [60] and clearly suggest that the tissue location of activation markers is an important factor in vaccine design.

Pre-clinical studies in NHP have an impediment in obtaining statistically significant results because of the small sample sizes that are practical, and the large individual and genetic differences among animals. These disadvantages were identified in our study, where the high level of inter-individual variation in the values of the immunological parameters in the vaccinated animals prevented the identification of subtle alterations related to the control group. Therefore, the absence of significant differences prevents us from reaching a clear conclusion about the role of these cells in DNA-*hsp65* vaccination.

Despite the lower magnitude of the immune cell and activation marker frequencies in the peripheral blood, our results suggest that our DNA-*hsp65* vaccination regimen is safe but provides limited ability to enhance peripheral cellular immune responses in the current vaccine format. Future preclinical trials with this vaccine delivered via EP may include a combination of a plasmid cytokine adjuvant and/or a protein prime–boost regimen, to help the induction of robust cellular immune responses.

## Data Availability

Data will be made available on request.

## Supplementary Materials

The following supporting information can be downloaded at: https://www.mdpi.com/article/10.3390/vaccines11121863/s1. Figure S1: Histopathological analysis at injection vaccine site. A focal and sparse mononuclear inflammatory process was seen in both side of the quadriceps muscle tissue was observed in all vaccinated animals (A and B); Table S1: HSP65 peptides pool design; Table S2: Follow-up profile of complete blood count in cynomolgus macaques vaccinated with electroporated *hsp6*5-DNA vaccine; Table S3: Follow-up profile of renal and hepatic markers in cynomolgus macaques vaccinated with electroporated-pVAX-hsp65DNA vaccine.
