# Supplementary material for: Safety and Immunogenicity of an In Vivo Muscle Electroporation Delivery System for DNA-hsp65 Tuberculosis Vaccine in Cynomolgus Monkeys
Source: Vaccines (Basel). 2023 Dec 18;11(12):1863. doi: 10.3390/vaccines11121863 (PMC10747856; doi:10.3390/vaccines11121863)
Supplement: Supplementary file 1 [file vaccines-11-01863-s001.zip › Figure S1.pdf]

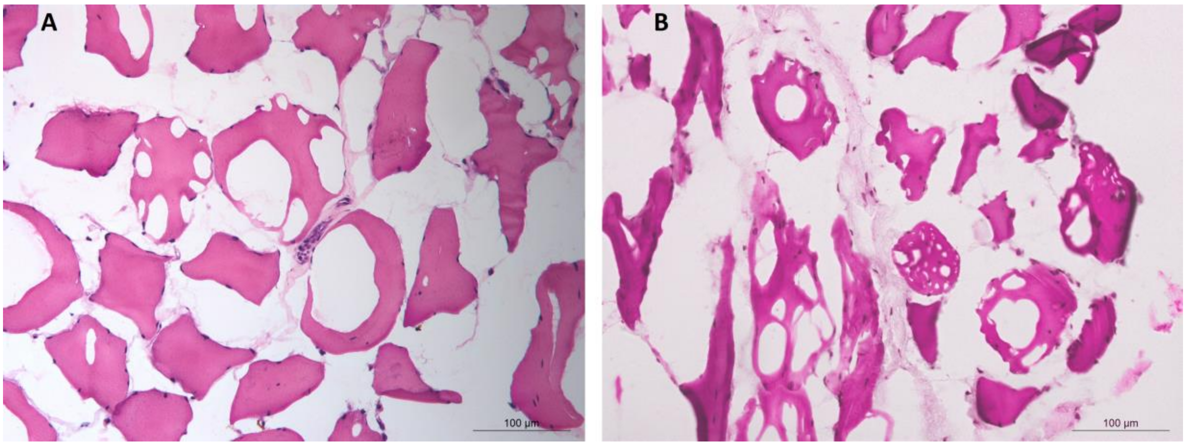

Figure S1: Histopathological analysis at injection vaccine site. A focal and sparse mononuclear inflammatory process was seen in both side of the quadriceps muscle tissue was observed in all vaccinated animals (A and B).
