# Supplementary material for: Safety and Immunogenicity of an In Vivo Muscle Electroporation Delivery System for DNA-hsp65 Tuberculosis Vaccine in Cynomolgus Monkeys
Source: Vaccines (Basel). 2023 Dec 18;11(12):1863. doi: 10.3390/vaccines11121863 (PMC10747856; doi:10.3390/vaccines11121863)
Supplement: Supplementary file 1 [file vaccines-11-01863-s001.zip › Supplem Table S3_ renal and hepatic profile.pdf]

Supplementary Table S3. Follow-up profile of renal and hepatic markers in cynomolgus macaques vaccinated with electroporated-pVAX-hsp65DNA vaccine

Time (months)	0		1		2		3		4		5		6	
	EP-pVAX-hsp65-DNA	HC	EP-pVAX-hsp65-DNA	HC	EP-pVAX-hsp65-DNA	HC	EP-pVAX-hsp65-DNA	HC	EP-pVAX-hsp65-DNA	HC	EP-pVAX-hsp65-DNA	HC	EP-pVAX-hsp65-DNA	HC
Urea	21.4±4.3	18.5±7.0	26.6±4.4	23.0±6.3	25.7±5.2	25.3±10.7	35.2±6.0	31.2±11.7	31.6±7.7	26.0±6.9	37.2±8.6	29.5±4.0	26.0±9.7	18.2±1.5
Creatinine	1.2±0.35	1.0±0.19	1.1±0.34	1.0±0.08	1.2±0.30	1.0±0.19	1.1±0.26	0.9±0.15	1.3±0.42	1.1±0.19	1.1±0.38	1.0±0.10	1.3±0.38	1.1±0.31
Glucose	71.7±14.2	60.5±22.1	58.6±7.0	61.7±13.9	85.0±17.3	76.0±17.3	71.1±8.6	68.2±10.5	84.9±14.9	75.0±20.0	81.6±15.7	71.5±20.7	84.0±13.6	79.5±16.9
Triglycerides	149±112.5	91±35.7	97±56.1	51±19.5	125±85.3	87±33.4	139±51.7	124±41.4	139±102.2	82±34.5	125±101.5	64±10.7	194±127.9	110±39.5
Cholesterol	116±30.2	125±18.4	124±30.8	130±20.2	127±32.7	131±23.9	128±31.3	132±28.6	120±23.8	131±22.4	132±26.6	143±20.5	145±32.4	148±35.1
Total Bilirubin	0.18±0.03	0.20±0.04	0.18±0.04	0.22±0.66	0.15±0.03	0.18±0.02	0.16±0.03	0.17±0.06	0.18±0.03	0.20±0.02	0.19±0.06	0.20±0.05	0.15±0.06	0.20±0.03
Total Protein	7.3±0.57	7.3±0.74	7.3±0.41	7.0±0.37	7.4±0.35	7.3±0.26	7.2±0.37	7.1±0.13	7.5±0.42	7.4±0.40	7.4±0.60	7.5±0.37	7.8±0.48	7.4±0.45
Albumin	3.1±0.32	3.3±0.19	3.3±0.29	3.40±0.12	3.2±0.28	3.4±0.08	3.1±0.22	3.3±0.15	3.3±0.26	3.4±0.15	3.4±0.37	3.6±0.13	3.4±0.38	3.4±0.45
AST	32.9±10.7	40.0±8.0	32.0±7.5	36.5±5.9	23.1±7.8	31.5±9.3	26.4±4.1	36.0±6.8	23.4±6.3*	32.0±9.0	25.4±9.7	29.7±7.4	27.9±10.9	35.5±8.7
ALT	37.1±8.1	36.7±5.4	29.4±4.6	34.0±6.8	29.6±4.6	33.0±6.9	30.9±4.4*	32.7±1.7	33.4±6.9	37.5±8.7	33.1±2.4	35.0±5.3	34.2±4.9	39.7±8.6

EP-pVAX- hsp65-DNA - electroporated-pVAX-heat shock protein 65 DNA vaccine; HC – Health control animals
