# Supplementary material for: Safety and Immunogenicity of an In Vivo Muscle Electroporation Delivery System for DNA-hsp65 Tuberculosis Vaccine in Cynomolgus Monkeys
Source: Vaccines (Basel). 2023 Dec 18;11(12):1863. doi: 10.3390/vaccines11121863 (PMC10747856; doi:10.3390/vaccines11121863)
Supplement: Supplementary file 1 [file vaccines-11-01863-s001.zip › vaccine-2701540 2nd version_Supplem Table S2_ hematological profile_2nd version.pdf]

Supplementary Table S2. Follow-up profile of complete blood count in cynomolgus macaques vaccinated with electroporated *hsp65*-DNA vaccine.

Time (days)	0		30		60		90		120		150		180	
	vaccine	HC	vaccine	HC	vaccine	HC	vaccine	HC	vaccine	HC	vaccine	HC	vaccine	HC
Erythrocytes (x10^6/ml)	6.7±0.6	6.5±0.2	6.5±0.6	6.3±0.3	6.4±0.6	6.2±0.3	6.6±0.6	6.4±0.5	6.6±0.5	6.5±0.3	6.6±0,6	6.5±0.3	6.6±0.6	6.4±0.7
Hematocrit (%)	42.±1.8	40.7±3.1	40.8±1.5	39.1±1.2	40.1±2.1	38.8±1.6	41.7±1.9	40.3±0.6	42.1±1.5	40.8±1.6	42.2±1.6	40.8±0.8	44.0±2.5	41.6±3.3
Hemoglobin (g/dl)	12.3±0.5	11.9±0.7	12.1±0.4	11.8±0.5	12.0±0.6	11.5±0.7	11.9±0.4	11.6±0.3	12.1±0.3	11.9±0.4	12.1±0.5	12.5±1.1	12.4±0.6	11.9±0.8
MCV (fl)	62.7±4.0	62.3±4.4	62.7±4.1	62.4±5	62.4±4.2	62.1±4.8	63.,8±3.7	63.2±5.5	63.8±3.5	62.9±4.5	63.9±3.7	62.6±4.3	66.5±3.9	65.0±4.7
MCHC (%)	29.2±0.5	29.4±1.0	29.7±0.5	30.1±1.2	29.6±0.6	29.8±1.5	28.7±0.7	28.9±1.1	28.8±0.4	29.2±1.0	28.8±0.5	29.4±1.1	28.3±0.5	28.6±0.6
Platelets (x10^3/ml)	475±124	378±87	418±85	351±47	416±80	332±42	424±94	358±26	375±59	303±40	386±112	329±17	428±89	316±43
Leucocytes (x10^3/ml)	11.4±4.4	7.9±2.4	10.3±2,.5	7.5±0.8	11.2±3.0	6.4±2.5	11.8±2.5	8.1±2.1	9.2±1.9	7.8±2.4	11.3±4.2	7.1±1.3	13.1±4.7	9.3±3.6
Lymphocytes (x10^3/ml)	4.2±2.0	2.6±2.2	4.8±0.8	3.6±1.8	4.8±0.9	3.7±1.1	4.3±0.9	3.2±2.0	3.9±0.7	3.2±1.4	4.7±2.2.3	2.8±1.6	4.9±2.2	3.8±1.8
Monocytes (x10^3/ml)	1.9±0.4	1.3±0.6	1.3±0.7	0.8±0.2	1.0±0.4	0.8±0.3	0.7±0.1	0.9±0.2	0.7±0.1	1.1±0.3	0.9±0.3	0.4±0.1	2.3±1.4	0.7±0.2
Granulocytes (x10^3/ml)	5.3±1.5	3.9±0.9	4.2±4.4	3.1±0.8	5.4±1.3	2.0±0.6	6.8±1.4	3.9±0.7	5.0±1.7	3.2±0.3	5.7±0.6	3.9±0.3	6.8±2.1	4.8±0.9

vaccine - electroporated *hsp65*-DNA; HC – Health control animals
